# Structural and Biochemical Changes in Salicylic-Acid-Treated Date Palm Roots Challenged with *Fusarium oxysporum* f. sp. *albedinis*

**DOI:** 10.4061/2011/280481

**Published:** 2011-12-07

**Authors:** Abdelhi Dihazi, Mohammed Amine Serghini, Fatima Jaiti, Fouad Daayf, Azeddine Driouich, Hassan Dihazi, Ismail El Hadrami

**Affiliations:** ^1^Laboratoire de Biotechnologies, Protection et Valorisation des Ressources Végétales, Faculté des Sciences Semlalia, Université Cadi Ayyad, B.P. 2390, Marrakech 40 001, Morocco; ^2^Laboratoire de Biotechnologie et de Valorisation des Ressources Naturelles, Faculté des Sciences, Université Ibn Zohr, Agadir 80060, Morocco; ^3^Department of Plant Science, University of Manitoba, Winnipeg, MB, Canada R3T 2N2; ^4^Laboratoire Glyco-MEV, Université de Rouen, 76821 Rouen, France; ^5^Clinical Proteomics Laboratories, University of Göettingen, 37075 Göttingen, Germany

## Abstract

Histochemical and ultrastructural analyses were carried out to assess structural and biochemical changes in date palm roots pretreated with salicylic acid (SA) then inoculated with *Fusarium oxysporum* f. sp. *albedinis* (Foa). Flavonoids, induced proteins, and peroxidase activity were revealed in root tissues of SA-treated plants after challenge by Foa. These reactions were closely associated with plant resistance to Foa. Host reactions induced after inoculation of SA-treated plants with Foa included the plugging of intercellular spaces, the deposition of electron-dense materials at the sites of pathogen penetration, and several damages to fungal cells. On the other hand, untreated inoculated plants showed marked cell wall degradation and total cytoplasm disorganization, indicating the protective effects provided by salicylic acid in treated plants.

## 1. Introduction

Fusarium wilts are economically important soil-borne diseases that result in significant crop losses and damage to natural ecosystems. Bayoud is a vascular wilt caused by *Fusarium oxysporum* f. sp. *albedinis* (Foa), and it is the most serious fungal disease threatening date palm plantations in North Africa, especially in Morocco. Resistance to the pathogen is found only in a few cultivars of date palm, which unfortunately produce poor-quality fruit [1].

A promising strategy for reducing diseases is based on the induction of resistance by challenging plants with defence elicitors. Plant defence mechanisms are represented by a number of structural and biochemical changes such as phytoalexin synthesis [2], callose deposition [3], cell wall lignification [4], and pathogenesis-related (PR) proteins induction [3]. However, these reactions do not always correlate with disease prevention. Among others, salicylic acid (SA) is known to be involved in regulating plant defences [5, 6]. Although the mechanism of SA-mediated plant defence is not completely understood in all crops, its central role in plant defence is well established [6–8]. Accumulation of SA has been shown to be crucial for the expression of PR proteins [9] which have antimicrobial activity and thought to be involved in plant resistance. Among them, peroxidases were described in plant defence against both necrotrophic and biotrophic pathogens [10]. Peroxidases can generate a physical barrier to prevent the penetration of the pathogen into the host tissues; it catalyzes the H_2_O_2_-dependent crosslinking of cell wall components leading to cell wall lignification [11].

In the date palm-Foa pathosystem, the first report confirming the involvement of SA in plant defence was by Dihazi et al. [12], who showed that application of SA activated the metabolism of phenolic compounds and induced the biosynthesis of some hydroxycinnamic acid derivatives then described as sinapic acid derivatives [13].

Flavonoids are also detected in plant cell walls in interactions with a resistance outcome [14], and their concentration increased significantly in clover roots in response to infection with *Pythium ultimum *[15]. In the date palm-Foa pathosystem, flavonoids have been described in the roots by Ziouti et al. [16] and were correlated to date palm callus defence against Foa [17].

Given the complexity of the root system of date palm plants, it is difficult to understand the role of the above-noted defence responses without a microscopic analysis of their effects in situ. Therefore, the objective of the present paper was to contribute a cytological investigation to studies on the mechanisms involved in SA-induced defence reactions. We investigated both structural and biochemical changes involved in conferring resistance to date palm cultivar Jihel against Foa after treatment with SA. These included induced phenolics (i.e., flavonoids) as well as induced activity of peroxidase. Furthermore, an ultrastructural analysis was carried out with the aim to elucidate the role of SA in events involved in resistance of date palm towards Foa.

## 2. Materials and Methods

### 2.1. Plant Inoculation with Foa and SA Treatment

We used 6-month-old date palm seedlings obtained from seeds produced by cultivar Jihel, highly susceptible to Foa wilt. They were grown in a greenhouse (16 h photoperiod) and then inoculated by injecting 10 *μ*L of Foa conidial suspension (6 × 10^6^ spores·mL^−1^) into the roots. The inoculum was prepared using the strain ZAG known for its aggressiveness [18]. Seedlings were treated with SA (100 *μ*M) by soaking their roots in an SA solution three days before infiltration with the inocula. Two groups of treated plants were considered, inoculated (TI) and uninoculated (TU). Control treatments were untreated inoculated (UI) and untreated uninoculated (UU) plants. Plant material was collected, 15 days after inoculation, from areas with necrotic lesions when disease symptoms became visible.

### 2.2. Staining of Proteins

Roots were fixed overnight at 4 ± 1°C in a solution of 2% glutaraldehyde, 2% paraformaldehyde, 1% caffeine, and 0.2 M phosphate buffer (pH 7.2). Tissues were dehydrated in a graded ethanol series and embedded in resin (Technovit7100). Sections (3 *μ*m) were double-stained in Periodic Acid-Schiff (PAS) reagent and Naphthol Blue-Black (NBB). Soluble and reserve proteins were specifically stained in dark blue with NBB [19] and PAS stains starch reserves and cell walls pink [20]. Images were examined by conventional light microscopy.

### 2.3. Peroxidase Activity

#### 2.3.1. Histolocalization of Peroxidase Activity

Histological staining for peroxidase activity was carried out using the protocol described by Gahan [21]. Sections from date palm root tissues were fixed for 1 hour in 4% paraformaldehyde in 0.2 M potassium phosphate buffer pH 7.2. Staining was done by incubation of the sections in Tris-HCl buffer 0.05 M, pH 7.6 containing 1.4 × 10^−3^ M diaminobenzidine (DAB), 2% H_2_O_2_ (1%), and 10^−1^ M 3-amino-1,2,4-triazole. Images were examined using conventional light microscopy.

#### 2.3.2. SDS-PAGE of Peroxidases

SDS-PAGE of peroxidases was carried out using acrylamide-bisacrylamide (30–0.8) gels as described by Baaziz [22]. Enzyme extraction was conducted with potassium phosphate buffer (pH 7.2) containing 4% polyvinylpyrrolidone. For each treatment, root tissues (200 mg Fresh Weight) of three seedlings were collected from areas with necrotic lesions and then homogenized in 1 mL of the buffer solution. The homogenate was centrifuged at 10 000 g for 30 min, and the supernatant was used as the enzyme extract. All steps were run at 4°C. Twenty *μ*L of enzyme extract were subjected to electrophoresis. Electrophoretic separation was carried out at constant current of 60 mA for 2-3 h. The electrophoresis buffer was TGS (Tris-Glycine-SDS) pH 8.1. Peroxidase activity was visualized in gels using diaminobenzidine and H_2_O_2_ in 0.2 M acetate buffer, pH 5. The reaction was stopped by removing the substrates and rinsing the gels with water. The SDS-PAGE experiments were repeated twice to confirm the reproducibility of the method.

### 2.4. Histolocalization of Phenolic Compounds

Small sections of date palm roots were embedded in 3% agarose. Transverse sections (30 *μ*m thickness) were cut using a freezing-stage microtome. After staining with the different reagents, the sections were mounted in the reagents solution. Sections were viewed using a light microscope equipped with a blue filter set at a 420 nm excitation and a 515–560 nm barrier filter. Neu's reagent [23], a standard reagent for phenolic compounds, was used. Transverse sections were immersed 2–5 min in 1% 2-amino-ethyldiphenylborinate (Fluka) in absolute methanol and then mounted in a glycerine-water (15/85, v/v) solution and examined using fluorescence microscopy. In the presence of Neu's reagent, the fluorescence of caffeic acid derivatives and flavonoids became greenish yellow and bright orange, respectively, under blue light.

### 2.5. Electron Microscopy

For transmission electron microscopy (TEM), samples were preserved by quick fixation in 3% (v/v) glutaraldehyde in a 0.1 M cacodylate buffer pH 7.2 for 12 h at room temperature. After rinsing them three times with the fixing cacodylate buffer, samples were post-fixed for 2 h at room temperature in darkness in 1% OsO4 prepared in cacodylate buffer. The samples were then dehydrated in a series of ethanol solutions to 100%, infiltrated, and embedded in Spurr's low viscosity epoxy resin [24]. Samples were processed using a Reichert-ultracut S microtome. Sections were contrasted with uranyl acetate for 30 min and lead citrate for 30 min in darkness. Analyses were carried out using a transmission electron microscope (Jeol 100 EX (Tokyo, Japan)).

### 2.6. Statistical Analysis

For the 4 groups of treatment (UU, UI, TU, TI), 13 sections were used for each group and experiment repeated twice. The percentage of sections that showed proteins, flavonoids, and peroxidase activity was calculated; significant differences were recorded between TI and UU treatments at *α* = 0.05 using chi-square test for goodness of fit (SPSS Statistics 17,0).

## 3. Results

### 3.1. SA Treatment and Symptomatology

**Figure 1:**
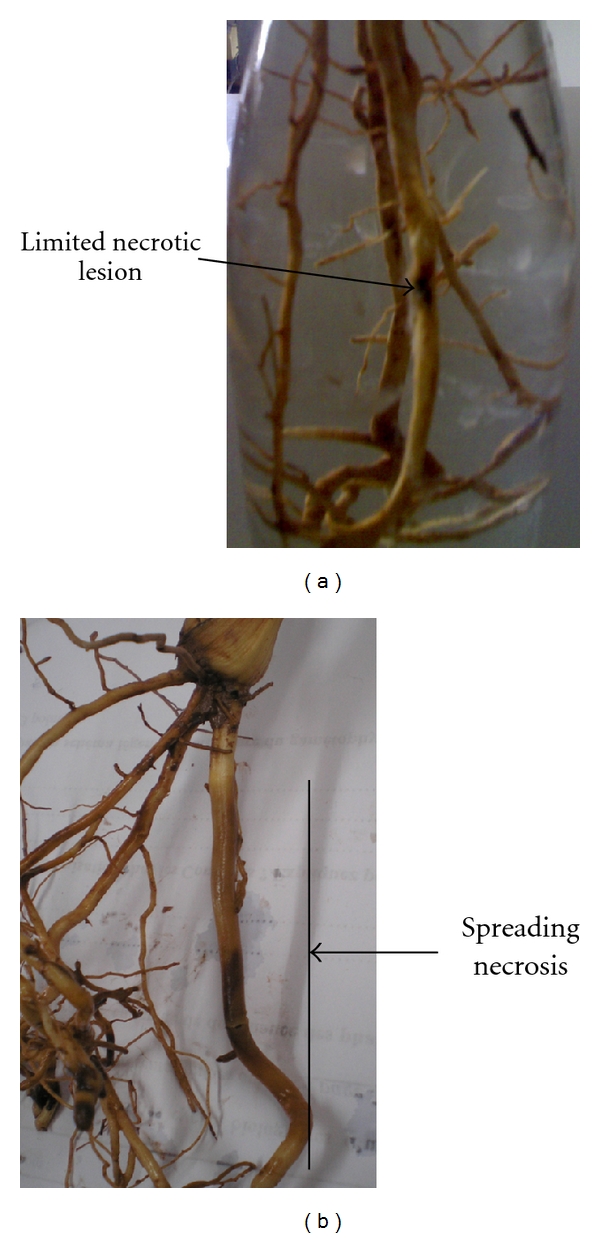
Without SA treatments, diseased control plants showed progressing wet necrosis of the roots (b). In contrast, after SA treatment, plants developed limited necrotic lesions (a).

Both SA-treated and nontreated plants were inoculated with *Fusarium oxysporum *f. sp. *albedinis* to determine their susceptibility to fungal attack. Typical disease symptoms, mainly characterized by the formation of enlarged brownish lesions at the sites of fungal contact, were visible by 15 d after inoculation (d.a.i.) in control plants. In contrast, with 100 *μ*M SA resulted in much less disease development than in nontreated date palm plants. Treated inoculated plants generally developed limited necrotic lesions, which reflected a delay in the colonization of the host plant by the pathogen (Figure 1).

### 3.2. Histological Observations of Infected Date Palm Root Tissues (Figure 2(b))

Observations of transversally sectioned root samples from nontreated plants that were inoculated with Foa showed that all tissues were massively invaded by the pathogen except for the sclerenchyma, which was seldom colonized. Root tissues were intensely colonized as evidenced by the presence of pathogen hyphae through the cortex, the endodermis, and the vascular stele.

### 3.3. Histochemical Features of SA-Induced Defence Reactions

#### 3.3.1. Staining of Induced Proteins

**Figure 2:**
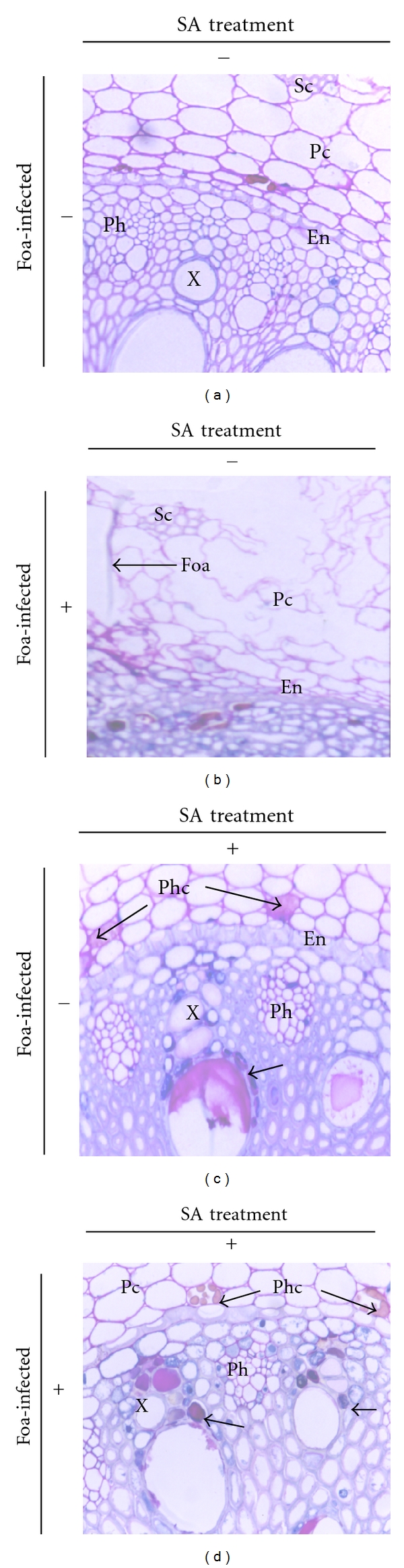
Light micrographs of samples from JHL date palm root tissues inoculated by *Fusarium oxysporum *f. sp. *albedinis* (Foa) (×40). Proteins were specifically stained in dark blue with NBB and PAS stains starch reserves and cell walls in pink. (a) Control roots (UU); (b) untreated and infected roots (UI); (c) SA-treated roots (TU); (d) SA-treated and Foa-infected roots (TI); note the presence of proteins at the cell layer surrounding the xylem vessels (arrow). X: xylem vessels; Ph: Phloem tubes; Pc: cortical parenchyma; Phc: Phenolic-storing cells.

Histochemical analysis confirmed the expression of induced proteins in SA-treated plants inoculated or uninoculated with Foa (Figures 2(c) and 2(d)). The proteins (stained in dark blue with NBB) were strongly expressed at the cell layer surrounding the xylem vessels and in some parenchyma cells near the endodermis. Control plants had impaired protein production (Figures 2(a) and 2(b)).

#### 3.3.2. Peroxidase Activity

**Figure 3:**
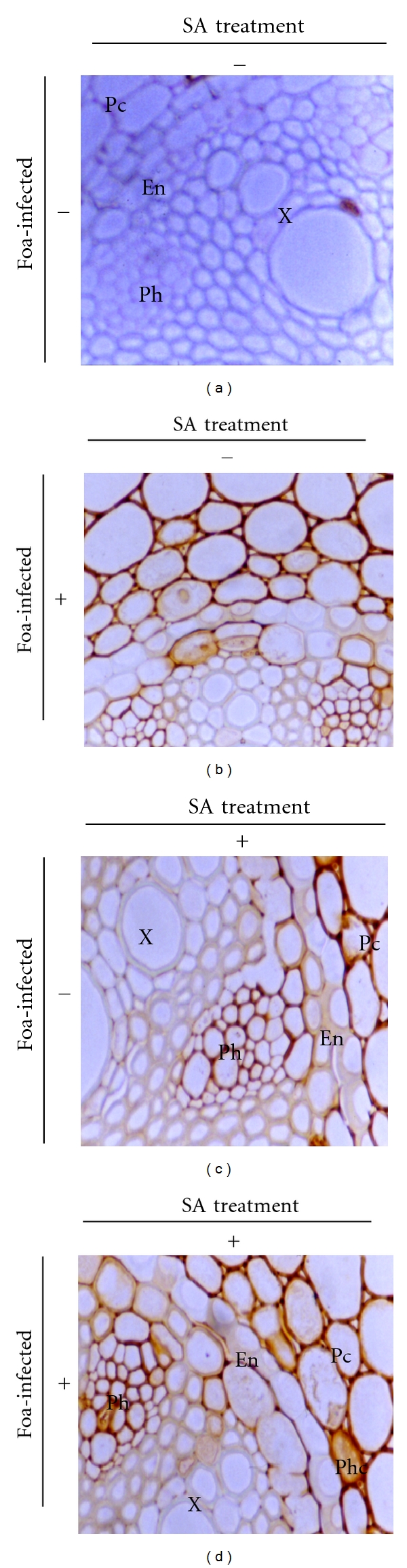
Cytochemical localization of peroxidase activity in date palm root tissues of JHL cultivar revealed by brown deposits after DAB staining. (a) control plants (UU; ×40); (b) infected plants (UI; ×60); (c) SA-treated roots (TU; ×60); (d) SA-treated and *Fusarium oxysporum *f. sp. *albedinis*-infected plants (TI; ×60). X: xylem vessels; Ph: phloem; En: endodermic cells; Pc: cortical parenchyma cells; Phc: Phenolic-storing cells.

**Figure 4:**
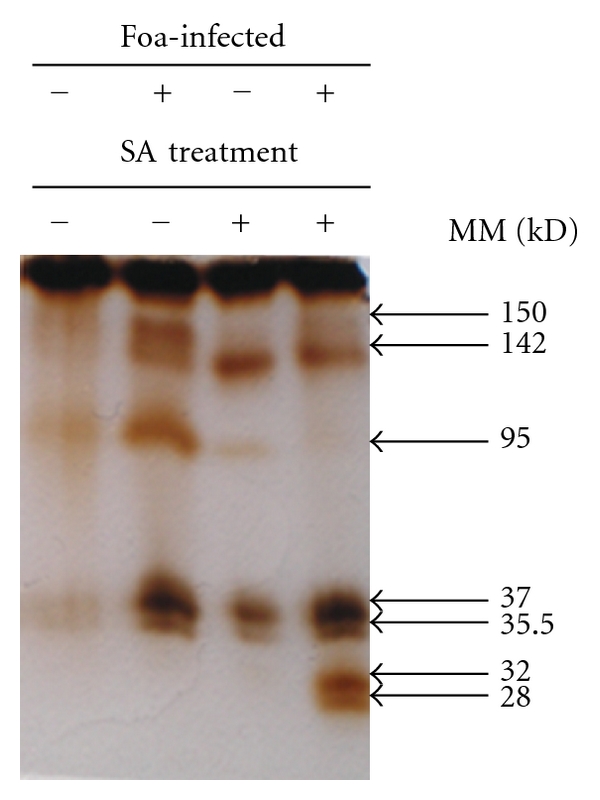
SDS-PAGE of peroxidase activity in roots of date palm Jihel cultivar visualized using DAB staining in response to treatment by salicylic acid (100 *μ*M) and inoculation with *Fusarium oxysporum* f. sp. *albedinis* at the 15th day after inoculation (RM: relative electrophoretic mobility).

Peroxidase activity revealed by brown deposits after DAB staining was noted in SA-treated plants both inoculated (85%) and uninoculated (60%) with Foa and also in untreated inoculated ones (70%). Such activity was localized in the cell walls and in the intercellular spaces of both the cortical parenchyma cells and the phloem tissues (Figures 3(b), 3(c), and 3(d)). In roots from control plants, such an activity was rather low (23%) (Figure 3(a)). In addition, SDS-PAGE analysis (Figure 4) confirmed this result; in Foa-infected plants two peroxidases isozymes with MM values of 150 and 142 kD were markedly expressed. These isozymes are present in trace amounts in untreated uninoculated plants and clearly decrease both in SA-treated and in SA-treated and inoculated ones. Interestingly, two additional isozymes with MM values of 32 and 28 kD were expressed only in SA-treated and inoculated plants and suggested that the activation of peroxidase with SA was more pronounced in infected plants. 

#### 3.3.3. Histochemical Staining of Phenolic Compounds

**Figure 5:**
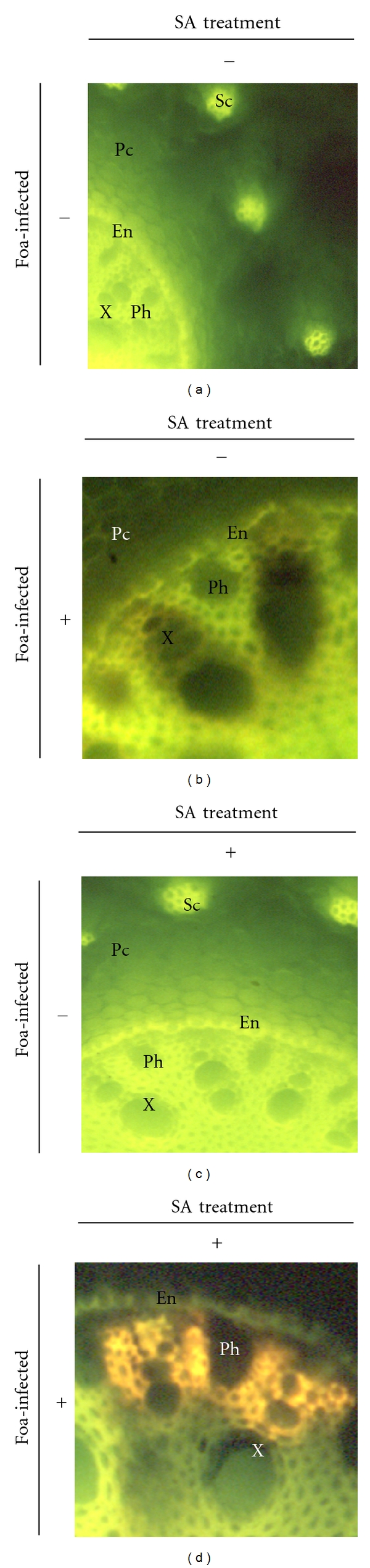
Histochemical localization of caffeic acid derivatives and flavonoids in date palm root tissues stained with Neu's reagent under blue light (green yellow and bright orange, resp.). (a: ×20) Control roots (UU), caffeic acid derivatives were observed at sclerenchyma, endodermis, and vascular parenchyma cell walls; (b: ×40) inoculated roots (UI), a dark zone appeared in cells situated near the xylem vessels. (c: ×20) SA treated roots (TU); (d: ×40) SA treated and *Fusarium oxysporum *f. sp. *albedinis-*infected roots (TI); note the presence of flavonoids in the vascular-parenchyma cell walls near the xylem vessels. X: xylem vessels; Ph: phloem; En; endodermic cells; Pc: parenchyma cells; Sc: sclerenchyma cells.

Neu's reagent is a standard histochemical reagent for many phenolic compounds. It stains flavonoids and caffeic acid derivatives bright orange and green yellow, respectively. It forms complexes with such phenolics which then emit a specific fluorescence. In date palm root controls treated with the Neu's reagent, a greenish yellow fluorescence was observed under blue light in sclerenchyma, endodermis, and vascular parenchyma cell walls. These caffeic acid derivatives revealed might be involved in lignification processes. When plants were inoculated with Foa, a decrease in this specific fluorescence was observed and a brownish color appeared in cells located near the xylem vessels (Figure 5(b)). When plants were treated with SA and challenged by Foa, flavonoids were induced (75%) (bright orange fluorescence) in the vascular-parenchyma cell walls near the xylem vessels (Figure 5(d)), probably at sites of potential pathogen penetration. Their presence might be associated with their involvement in plant defence.

#### 3.3.4. Ultrastructural Features of Infected Date Palm Roots and SA-Induced Defence Reactions

**Figure 6:**
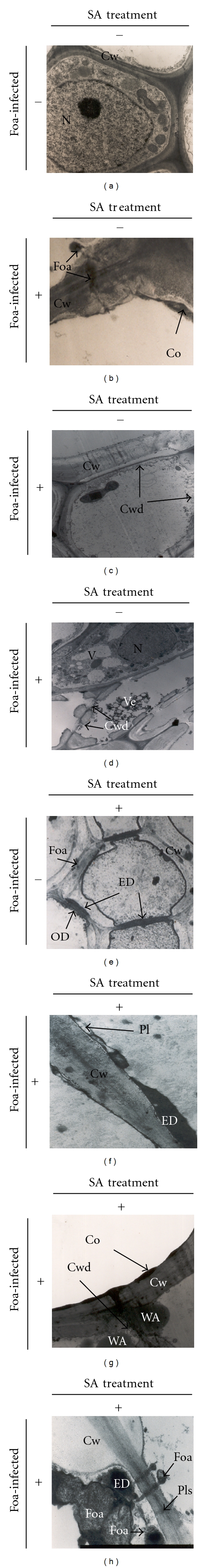
Transmission electron micrographs of date palm root tissues treated with SA and infected by *Fusarium oxysporum* f. sp. *albedinis* (Foa). (a: ×5000) Preserved cell wall (Cw) in control roots (UU); (b: ×20000), (c: ×6000), (d: ×5000) Untreated inoculated roots (UI); note the marked cell wall degradation (c) as well as the alteration of the cytoplasm, which was reduced to vesicular remnants (d). (e: ×6000), (f: ×12000), (g: ×12000), (h: ×30000) SA-treated and infected plants; note the accumulation of the electron-dense material (ED) (f), which forms a coat (Co) in the vessel lumen (g) and osmiophilic droplets (OD) at sites of intercellular spaces of parenchyma cells (e). Note also the wall appositions (WA) (g) and the disruption of the Foa hypha near the electron-dense material (ED) (h). Cw: cell walls; Ve: vesicles; Co: coat; Cwd: cell wall degradation; Pl: plasmalemma; Pls: plasmodesmata; N: nuclei; Foa: *Fusarium oxysporum* f. sp. *albedinis*.

Close examination of infected roots' ultrastructure showed that pathogen ingress toward the vascular stele coincided with extensive vascular disorganization and marked cell alterations of the cytoplasm which was frequently reduced to vesicular remnants (Figure 6(d)). The colonization was characterized by a marked deterioration of the cell walls resulting in its complete dissolution (Figure 6(c)) as compared to the preserved cell walls in uninoculated roots (Figure 6(a)). In all cases, the observed pattern of fungal colonization and host cell disorganization coincided with the occurrence of macroscopically visible symptoms along the roots, leading to severe plant wilting and eventually plant death. Fungal growth was mainly intracellular, but it could also occur intercellularly through direct host wall penetration. The germ-tube attempts to cross the host cell walls occurred preferably through the plasmodesmata at pit fields lacking secondary wall material (Figures 6(b) and 6(h)).

In SA-treated plants, the colonization by Foa differed markedly from that observed in control plants. Fungal growth was reduced, and hyphae were seldom seen in the tissues of the host roots. One of the most striking changes observed in SA-treated plants as compared with controls was the elaboration of several host defence reactions that included the deposition of an electron-dense material closely associated with the cell walls (Figure 6(f)). This material forms a coat in the vessel lumen (Figure 6(g)) and at sites of intercellular spaces leading to plug them (Figure 6(e)) and eventually to alter the invading hyphae (Figure 6(h)). Wall appositions were also observed at sites of pathogen invasion (Figure 6(f)). These structures were associated with fungal cell walls' distortion, breakdown of the plasma membrane, and an apparent densification of the cytoplasm (Figure 6(h)). They are probably meant to strengthen the host cell walls and to prevent the pathogen from spreading.

## 4. Discussion

We have previously described the role of SA in enhancing the survival of Foa-infected date palm plants by triggering the synthesis of new phenolic compounds [12] which were identified as hydroxycinnamic acid derivatives [13, 25]. treatment To our knowledge, this is the first report showing the effects of SA on the cytochemical changes of the root tissues in the date palm-Foa interaction. These results suggest that SA induced protein synthesis in both inoculated and uninoculated plants, especially in the cell layer surrounding the xylem vessels and also in vascular parenchyma cells located near the endodermis and in some cortical parenchyma cell walls. The proteins induced in response to SA treatment were strongly expressed in asymptomatic date palm plants and could play a role in resistance. In nontreated inoculated plants, these proteins were present, but not enough to stop the spreading of the pathogen and several signs of tissue alterations were noted.

In agreement with earlier studies [12], the present results confirmed that defence reactions in SA-treated plants were more pronounced after fungal challenge. Moreover, we have shown that both SA-treated plants and the infected ones have expressed high peroxidase activity, with two isozymes (150 and 142 kD) in inoculated plants that disappear in SA-treated ones and two new isozymes (32 and 28 kD) expressed only in SA-treated and inoculated plants. The expression of these new isozymes only in inoculated and SA-treated plants may constitute a sort of “priming” of date palm plants toward its pathogen.

This corroborates other findings that correlated date palm resistance against Foa with peroxidase activity [26–28]. Indeed, Jaiti et al. [28] had shown that jasmonic acid increased the peroxidase activity in date palm plants after Foa infection. Peroxidases are frequently responsive to SA or jasmonic acid [29], and it is widely known that they play a central role in host plant defences against pathogens [10]. Peroxidases may be involved in the peroxidation of substrate molecules, leading to the accumulation of highly toxic compounds, which may contribute to resistance via their antifungal potential [30]. Interestingly, a high peroxidase activity was detected in the phenolic-storing cells, especially in SA-treated date palm roots.

Furthermore, salicylic acid is known to repress some jasmonic acid pathway-related defence mechanisms [31, 32]. This may explain the presence of isozymes in inoculated plants then their absence after SA treatment. Why some isoforms of peroxidase are induced while others are repressed remains to be determined.

In addition, histochemical analysis showed that flavonoids accumulated only in SA-treated and Foa-inoculated plants and filled the vascular parenchyma cell walls. This confirms the implication of SA in sensitizing date palm plants to respond faster to its pathogen. The involvement of flavonoids in plant defence reactions is well documented [33]. These phenolics are different from those revealed in date palm root controls which were caffeic acid derivatives. Flavonoids were considered as important phenolic compounds detected in plant cell walls in resistant interactions [14]. Their accumulation has occurred only in the resistant cotton variety inoculated with the pathogen *Xanthomonas axonopodis *pv. *Malvacearum *[14]. More recently, Carlsen et al. [15] have shown that the concentrations of flavonoids formononetin and daidzein increased significantly in clover roots in response to infection with *Pythium ultimum*. Dixon [34] confirmed the antimicrobial action of flavonoids during plant-fungal pathogen defence responses. McNally et al. [35], using the *Podosphaera xanthii*-cucumber interaction, have also localized the production of C-glycosyl flavonoids in resistant plants. More recently, El Hadrami et al. [36] have shown the involvement of rutin in the interaction between potato and *Verticillium dahliae*.

In the date palm-Foa pathosystem, flavonoids have been detected in trace amounts in date palm roots [16] and have been suggested to play a role in date palm callus defence against Foa [17], but none of the previously published studies has reported the involvement of SA in triggering the accumulation of flavonoids related to date palm plant defence. Our data suggest that flavonoids were expressed after SA treatment in the cell walls of vascular parenchyma cells closely associated with the xylem vessels especially in tissues around the necrotic zone and this is associated with plant resistance. Dai et al. [37] have reported that the necrosis formations may come from the action of peroxidase on flavonoids during the lignification process.

To investigate whether the observed biochemical changes correlate with the structural modifications, date palm root tissues were examined at the ultrastructural level. In nontreated date palm plants, all root tissues were strongly colonized by Foa. Pathogen ingress into the vascular stele was characterized by severe host cell alterations, including cell wall degradation and alteration of the cytoplasm, which was frequently reduced to vesicular remnants. This may suggest that the fungus produces cell wall hydrolytic enzymes that hydrolyze host components and lead to fungal spread into the root tissues. Such enzymes were previously described in date palm-Foa interaction and were correlated to disease development [38].

The protection against Foa in SA-treated date palm plants was associated with a deposition of an electron-dense material, a wall apposition in host cells, and an increase in damaged fungal cells. This electron-dense material was closely associated with intercellular spaces, known to be strategic sites for pathogen spread. Such host reactions, obviously designed to halt pathogen ingress, were never seen in control plants. Fungal cells, near these electron-dense materials, were frequently disorganized, suggesting a fungitoxic environment generated by this material [39]. The nature of this material is not yet determined. Its reactivity to osmium tetraoxide indicates that these compounds are possibly of phenolic nature [40]. The accumulation of phenolic compounds in the host cell walls may contribute to enhancing the mechanical strength of these defensive barriers. According to our cytochemical observations, the electron-dense material formed in SA-treated root tissues could include flavonoids. Interestingly, in banana roots attacked by the nematode *Radopholus similis*, electron-dense materials have been associated with the impregnation of cell walls by flavonoids previously detected by histochemical analysis [41]. The same result was found in cotton leaves infected by *Xanthomonas campestris *pv. malvacearum [14]. The nature of the wall apposition formed after SA treatment is also not yet determined, but their involvement in pathogen attack was demonstrated since fungal cells near these wall appositions were altered. Several reports indicated that wall appositions contain callose and cellulose, as revealed by cytochemical analysis [42]. Such cellular changes led to several barriers preventing pathogen spread in the root tissues.

## 5. Conclusion

During this study, conclusive results were obtained. They provide evidence that SA treatment induces structural and biochemical changes in date palm roots in response to fungal infection. Cortical and vascular-parenchyma cells are the main cells involved in SA-induced response leading to the accumulation of flavonoids and other phenolic compounds, as well as the induction of new isomers of peroxidase. This confirms the possible implication of SA in triggering date palm defence against its pathogen and confers a promising strategy for effective control of the Bayoud disease.
